# A Systems Genetics Approach Identifies *CXCL14*, *ITGAX*, and *LPCAT2* as Novel Aggressive Prostate Cancer Susceptibility Genes

**DOI:** 10.1371/journal.pgen.1004809

**Published:** 2014-11-20

**Authors:** Kendra A. Williams, Minnkyong Lee, Ying Hu, Jonathan Andreas, Shashank J. Patel, Suiyuan Zhang, Peter Chines, Abdel Elkahloun, Settara Chandrasekharappa, J. Silvio Gutkind, Alfredo A. Molinolo, Nigel P. S. Crawford

**Affiliations:** 1 Genetics and Molecular Biology Branch, National Human Genome Research Institute, NIH, Bethesda, Maryland, United States of America; 2 Center for Biomedical Informatics and Information Technology, National Cancer Institute, NIH, Rockville, Maryland, United States of America; 3 Computational and Statistical Genomics Branch, National Human Genome Research Institute, NIH, Bethesda, Maryland, United States of America; 4 Medical Genomics and Metabolic Genetics Branch, National Human Genome Research Institute, NIH, Bethesda, Maryland, United States of America; 5 Cancer Genetics and Comparative Genomics Branch, National Human Genome Research Institute, NIH, Bethesda, Maryland, United States of America; 6 Oral & Pharyngeal Cancer Branch, National Institute of Dental and Craniofacial Research, NIH, Bethesda, Maryland, United States of America; University of Washington, United States of America

## Abstract

Although prostate cancer typically runs an indolent course, a subset of men develop aggressive, fatal forms of this disease. We hypothesize that germline variation modulates susceptibility to aggressive prostate cancer. The goal of this work is to identify susceptibility genes using the C57BL/6-Tg(TRAMP)8247Ng/J (TRAMP) mouse model of neuroendocrine prostate cancer. Quantitative trait locus (QTL) mapping was performed in transgene-positive (TRAMPxNOD/ShiLtJ) F2 intercross males (n = 228), which facilitated identification of 11 loci associated with aggressive disease development. Microarray data derived from 126 (TRAMPxNOD/ShiLtJ) F2 primary tumors were used to prioritize candidate genes within QTLs, with candidate genes deemed as being high priority when possessing both high levels of expression-trait correlation and a proximal expression QTL. This process enabled the identification of 35 aggressive prostate tumorigenesis candidate genes. The role of these genes in aggressive forms of human prostate cancer was investigated using two concurrent approaches. First, logistic regression analysis in two human prostate gene expression datasets revealed that expression levels of five genes (*CXCL14*, *ITGAX*, *LPCAT2*, *RNASEH2A*, and *ZNF322*) were positively correlated with aggressive prostate cancer and two genes (*CCL19* and *HIST1H1A*) were protective for aggressive prostate cancer. Higher than average levels of expression of the five genes that were positively correlated with aggressive disease were consistently associated with patient outcome in both human prostate cancer tumor gene expression datasets. Second, three of these five genes (*CXCL14*, *ITGAX*, and *LPCAT2*) harbored polymorphisms associated with aggressive disease development in a human GWAS cohort consisting of 1,172 prostate cancer patients. This study is the first example of using a systems genetics approach to successfully identify novel susceptibility genes for aggressive prostate cancer. Such approaches will facilitate the identification of novel germline factors driving aggressive disease susceptibility and allow for new insights into these deadly forms of prostate cancer.

## Author Summary

Prostate cancer is a remarkably common disease, and in 2014 it is estimated that it will account for 27% of new cancer cases in men in the US. However, less than 13% those diagnosed will succumb to prostate cancer, with most men dying from unrelated causes. The tests used to identify men at risk of fatal prostate cancer are inaccurate, which leads to overtreatment, unnecessary patient suffering, and represents a significant public health burden. Many studies have shown that hereditary genetic variation significantly alters susceptibility to fatal prostate cancer, although the identities of genes responsible for this are mostly unknown. Here, we used a mouse model of prostate cancer to identify such genes. We introduced hereditary genetic variation into this mouse model through breeding, and used a genetic mapping technique to identify 35 genes associated with aggressive disease. The levels of three of these genes were consistently abnormal in human prostate cancers with a more aggressive disease course. Additionally, hereditary differences in these same three genes were associated with markers of fatal prostate cancer in men. This approach has given us unique insights into how hereditary variation influences fatal forms of prostate cancer.

## Introduction

Prostate cancer is a common disease, and it is estimated that approximately 233,000 new cases will be diagnosed in in the United States alone in 2014 [1]. However, it typically runs an indolent course, with most men succumbing to unrelated diseases. This is reflected in the low prostate cancer-specific mortality, with ∼29,000 men dying from this disease in the same period in the US. Currently, the assessment of prognosis relies heavily upon the evaluation of traditional clinical and pathological variables, and is fraught with inaccuracies. These inaccuracies lead to over-treatment of prostate cancer, which causes unnecessary suffering resulting from aggressive therapeutic interventions, and represents a significant public health burden. Accordingly, there is a pressing need to improve the molecular characterization of prostate cancer, in order to facilitate an improved prognostic accuracy and to detect men at increased risk of developing aggressive, fatal forms of this disease.

One such feature that is garnering increased attention is the emergence of prostate tumors with a neuroendocrine (NE) phenotype [2]. Small cell NE prostate carcinoma is a rare histological subtype, which comprises 0.3% to 1.0% of all prostate malignancies [3]. Compared to prostate adenocarcinoma, which is the most common histological subtype, it typically runs a more aggressive course and is associated with visceral metastasis and poor outcomes (median survival  = 10.0 months *vs*. 125.0 months for adenocarcinoma) [4]–[6]. However, it is becoming increasingly apparent that prostate adenocarcinomas, which comprise 90–95% of all prostatic neoplasms [3], with extensive NE characteristics are associated with a particularly poor prognosis. Specifically, autopsy studies have demonstrated that at least 20–30% of end-stage prostate adenocarcinomas exhibit a significant degree of NE differentiation (NED) [7], [8]. Furthermore, this NE phenotype is particularly prevalent in patients treated with androgen deprivation therapy (ADT), and the appearance of recurrent tumors with NE characteristics following ADT is associated with castrate resistance, visceral metastasis, and death [8], [9]. In addition, the incidence of prostate carcinomas with a prominent NE phenotype is expected to increase as use of second generation ADTs (e.g., enzalutamide, abiraterone) becomes more widespread, such that NED will likely represent a new mechanism of therapeutic resistance [10].

The pathogenesis of NED remains unclear. Recent studies have demonstrated that *RB1* loss is a crucial element of the pathogenesis of NE prostate cancer [10]. Additionally, these tumors are often associated with loss of androgen receptor expression, activation of the PI3K pathway, and amplification of *N-MYC* and *AURKA* [2]. However, like all forms of prostate cancer, the initiation and progression of NED will be influenced by host germline variation. Genome-wide association studies (GWAS) have revolutionized our understanding of how heritable factors influence prostate cancer development, and have facilitated the identification of multiple loci associated with aggressive disease (e.g., [11]). Yet GWAS have not been able to explain the complete influence of heritability on disease susceptibility. Therefore, alternative approaches for defining susceptibility will be required to augment GWAS and to fully understand how the germline modifies susceptibility to aggressive phenotypes like NED.

**Figure 1:**
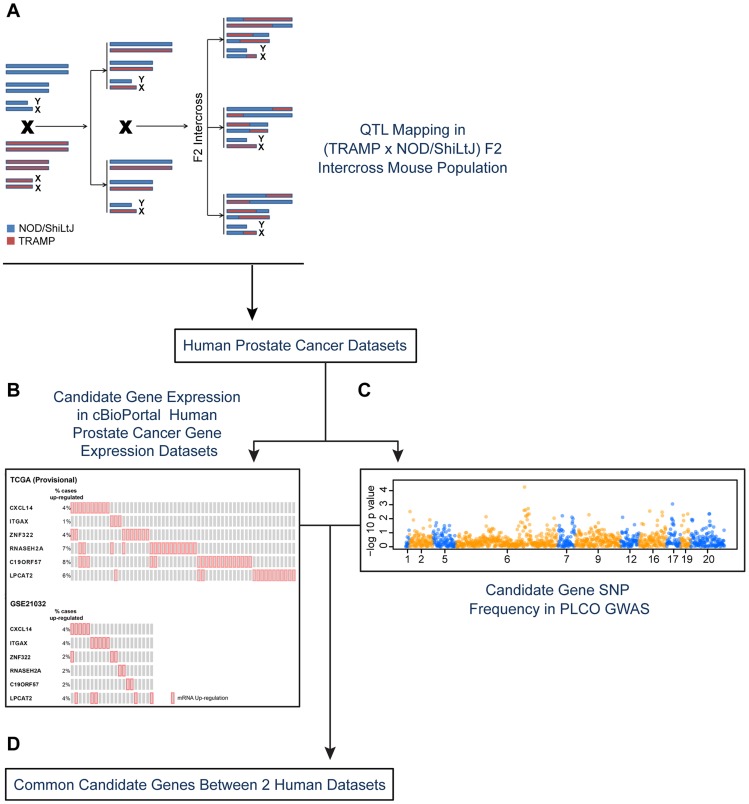
Experimental strategy for identifying novel susceptibility genes for aggressive prostate cancer. Candidate aggressive disease modifier genes were identified in an F2 intercross population involving the TRAMP mouse model of prostate tumorigenesis and the NOD/ShiLtJ strain of mouse, which is highly susceptible to aggressive disease development (A). This strategy involved QTL mapping to identify genomic regions associated with aggressive disease traits, followed by eQTL mapping and gene expression-trait correlation analyses to nominate candidate modifiers. Following this, a strategy involving two types of data derived from human prostate patients was used to nominate the highest priority candidate genes: (B) human prostate cancer primary tumor gene expression datasets; and (C) a human prostate cancer GWAS dataset. Only those genes designated as being associated with aggressive disease development in both the tumor gene expression and GWAS datasets were designated as being high priority candidate genes (D).

The work presented here utilizes a systems genetics approach, which involves the integration of lines of evidence from a mouse model of aggressive prostate cancer and several human prostate cancer datasets to identify novel genes associated with aggressive disease (Figure 1). Candidate genes are initially identified using the C57BL/6-Tg(TRAMP)8247Ng/J (TRAMP) mouse model of neuroendocrine prostate cancer, which develops extensive tumorigenesis and metastasis by 30 weeks of age [12]–[14]. Our earlier work demonstrated that disease aggressiveness in the TRAMP mouse is substantially modified by host genetic background [15]. This earlier study involved performing a ‘strain survey’ experiment where wildtype TRAMP mice were bred to one of eight inbred strains of mice. Characterization of disease aggressiveness traits in the eight resulting F1 strains revealed substantial strain-specific differences in prostate tumorigenesis and metastasis. Since the SV40 T antigen was expressed at equal levels and at the same developmental time point in each of the eight F1 strains, we concluded that the observed phenotypic differences in disease aggressiveness were a consequence of germline variation [15].

To explore the origins of this, an F2 mapping panel involving TRAMP and NOD/ShiLtJ, which is a strain that is highly susceptible to developing aggressive tumorigenesis, was generated. These mice were used to map quantitative trait loci (QTLs) associated with aggressive NE prostate cancer. Following this, QTL candidate genes were nominated from microarray gene expression data derived from (TRAMP × NOD/ShiLtJ) F2 tumors through a combination of expression QTL (eQTL) mapping and gene expression-trait correlation analysis. The relevance of these QTL candidate genes to aggressive forms of human prostate cancer were explored through two concurrent approaches: first, by correlating their expression levels with disease free survival (DFS) in two prostate tumor gene expression cohorts; and second, by analyzing a human GWAS dataset to correlate the frequencies of QTL candidate gene single nucleotide polymorphisms (SNPs) with clinical markers of disease aggressiveness. This approach, which is novel to the field of prostate cancer to the best of our knowledge, facilitated the identification of three novel aggressive prostate cancer susceptibility genes: *CXCL14*, *ITGAX*, and *LPCAT2*.

## Results

### Aggressive Prostate Cancer-Associated Traits in (TRAMP × NOD/ShiLtJ) F2 Mice

Earlier work demonstrated that germline variation present in the NOD/ShiLtJ strain renders (TRAMP × NOD/ShiLtJ) F1 male mice significantly more susceptible to aggressive prostate tumorigenesis [15]. Specifically, (TRAMP × NOD/ShiLtJ) F1 males displayed significantly increased primary tumor burden, local metastasis to regional lymph nodes, and distant metastasis to visceral organs including the lung, liver and kidneys compared to wildtype TRAMP C57BL/6J mice. Therefore, we hypothesized that the introduction of germline polymorphism through breeding will allow for the mapping of QTLs associated with aggressive tumorigenesis in the TRAMP mouse.

To investigate this hypothesis, a (TRAMP × NOD/ShiLtJ) F2 intercross population consisting of 228 transgene-positive males was developed. Mice were aged until 30 weeks of age or until humane endpoints were achieved. As expected, substantial variation in aggressive prostate cancer phenotypes was observed in these F2 mice (Table S1). Of particular note, it was clear that there was a strong level of interdependency between tumor related-traits (primary tumor burden, seminal vesicle tumor burden) and traits commonly associated with survival in human prostate cancer (reviewed in [16]; e.g., age at death, distant metastasis free survival [DMFS], presence or absence of lymph node metastasis, lymph node metastasis burden; Figure S1) in the F2 mapping population. As would be expected in humans, larger primary tumors were positively correlated with a younger age of death, a substantially reduced DMFS, an increased risk of lymph node metastasis, and an increased lymph node metastasis burden (Figure S1A–D). The converse, however, was true for seminal vesicle tumor burden, which was negatively correlated with the same traits (Figure S1E–H). Accordingly, there was a significant negative correlation between primary tumor burden and seminal vesicle tumor burden (Pearson's r = −0.41, *P* = 7.40×10^−11^; Figure S2). Earlier work with the TRAMP model has demonstrated that these seminal vesicle tumors represent a form of epithelial-stromal tumor that resemble phyllodes tumors [17], [18], which are an uncommon neoplasm of uncertain malignant potential in humans [19]. However, our data clearly demonstrate that mice with greater seminal vesicle tumor burden, and thus a lower primary tumor burden, are less prone to more aggressive disease forms. Therefore, the germline polymorphisms driving lower seminal vesicle tumor burden and higher primary tumor burden may be associated with a predisposition for more aggressive disease.

### Multiple QTLs are Associated with Aggressive Tumorigenesis in (TRAMP × NOD/ShiLtJ) F2 Mice

QTLs were mapped in (TRAMP × NOD/ShiLtJ) F2 males by performing a genome scan using 666 informative SNPs. Analyses were performed in J/qtl [20] using a single-locus model of inheritance. QTLs were considered statistically significant when genome-wide α<0.05. For metastasis-related traits, a total of four QTLs were observed: two for DMFS (chromosome 1 [logarithm of odds score (LOD)  = 3.93] and chromosome 11 [LOD  = 3.97]); one for lymph node metastasis burden on chromosome 13 (LOD  = 4.69); and one on chromosome 11 for liver surface metastasis count (LOD  = 4.01). A total of five QTLs were observed for tumor-related traits: one for primary tumor burden on chromosome 13 (LOD  = 4.86); and four for seminal vesicle tumor burden (chromosome 2 [LOD  = 5.01]; chromosome 4 [LOD  = 5.24]; chromosome 8 [LOD  = 4.22]; and chromosome 17 [LOD  = 5.20]). Finally, two QTLs were evident for age of death, on chromosome 7 (LOD  = 4.35) and chromosome 8 (LOD  = 4.65). QTL data are summarized in Table 1 and Figure S3. As is typical with F2 intercross populations, the confidence intervals of QTLs, as defined by the 2-LOD drop beyond the peak region of linkage, are broad, and each QTL will encompass many hundreds of genes. Additionally, it should be noted that these eleven QTLs in fact represent nine genomic regions with overlap of age of death and seminal vesicle tumor burden QTLs on chromosome 8, and nodal metastasis burden and primary tumor burden loci on chromosome 13.

**Table 1:** QTLs identified in (TRAMP × NOD/ShiLtJ) F2 mice.

Phenotype	Chromosome	LOD Score	*P*_genome_	Peak Linkage (cM)	2-LOD Confidence Interval (bp)	
					Start	End
Distant Metastasis-Free Survival						
	1	3.93	0.042	35.0	40,760,231	95,290,730
	11	3.97	0.039	30.9	41,325,431	69,191,538
Nodal Metastasis Burden						
	13	4.69	0.011	22.1	4,829,663	46,774,063
Liver Surface Metastasis Count						
	11	4.01	0.037	8.6	11,062,569	35,356,130
Prostate Tumor Burden						
	13	4.86	0.007	18.7	4,758,113	60,501,553
Seminal Vesicle Tumor Burden						
	2	5.01	0.005	84.4	146,404,042	165,979,416
	4	5.24	0.003	7.6	5,191,558	53,264,210
	8	4.22	0.022	52.8	83,633,294	111,798,566
	17	5.20	0.004	11.1	3,499,649	36,093,828
Age of Death						
	7	4.35	<0.001	76.4	122,268,816	144,131,415
	8	4.65	<0.001	50.8	87,425,863	111,798,566

### QTL Candidate Gene Nomination through Microarray Analysis of F2 Tumors

Integration of germline variation and transcriptome data is a well-established means of nominating QTL candidate genes that influence a given trait through expression-related mechanisms [21], [22]. Specifically, QTL candidate gene transcripts identified through this approach will possess both of the following: 1) they will exhibit a proximal expression QTL (eQTL), which we define as an eQTL mapping ≤1 megabase (Mb) upstream or downstream of the transcription start site since 95% of enhancers are predicted to target transcripts within this range [23]; and 2) their expression levels will be correlated with the trait of interest. Only the expression of transcripts physically located within QTLs were considered in these analyses. We hypothesize that QTL candidate genes modifying susceptibility to aggressive prostate tumorigenesis through transcriptional-related mechanisms in (TRAMP × NOD/ShiLtJ) F2 males will possess both of these characteristics.

**Figure 2:**
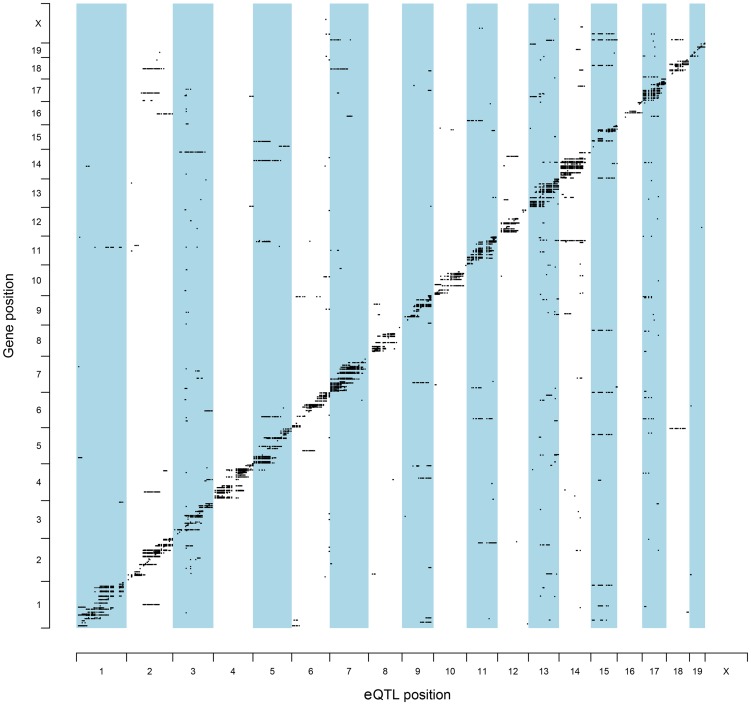
Genomic locations of eQTLs relative to their cognate transcript. The chromosomal locations for all statistically significant eQTLs identified in (TRAMP × NOD/ShiLtJ) F2 tumors (FDR <0.05) are illustrated relative to their associated transcript.

To identify QTL candidate genes in this manner, microarray analysis was performed to analyze patterns of global gene expression in all available F2 prostate tumors (n = 126). Expression QTL mapping was performed using Matrix eQTL [24]. Benjamini-Hochberg false discovery rates (FDR) were calculated to correct for multiple testing [25], with an FDR <0.05 used as the threshold for significant eQTLs. A total of 9,510 eQTLs were evident in TRAMP × NOD F2 tumors, of which 854 were defined as proximal eQTLs (Table S2) and 8,656 as distal and/or trans-eQTLs (Table S3). However, of the 8,656 distal and/or trans-eQTLs, only 1,560 associations were between a SNP and transcript on different chromosomes (i.e., a true *trans*-eQTL). The high number of distal eQTLs, which reside on the same chromosome as the cognate transcript but outside of the 1 Mb window for mapping proximal eQTLs most likely reflects the low level of recombination typically observed in F2 populations. The genomic distributions of eQTLs relative to their cognate transcript are illustrated in Figure 2. Of the 854 proximal eQTLs identified, 147 resided within the 2-LOD confidence intervals of the eleven aggressive disease QTLs described in Table 1.

To further increase the stringency of QTL candidate gene identification, the expression levels of all transcripts within the boundaries of each of 11 aggressive disease QTLs were correlated with the QTL trait (Tables S4–S14). Using the Benjamini-Hochberg FDR method [25] to correct for multiple testing (FDR <0.05), 35 high-confidence QTL candidate genes were identified, each of which exhibited a statistically significant proximal eQTL and correlation between transcript expression and the trait of interest (Table 2).

**Table 2:** QTL candidate genes identified in (TRAMP × NOD/ShiLtJ) F2 mice.

QTL		PROXIMAL eQTL ANALYSIS							CORRELATION ANALYSIS			Founder Strain eQTL Allele Correlated with Increased Expression	QTL Candidate Gene	Human Ortholog
Phenotype	Chr	eQTL	Position (bp)	Expressed Transcript	β	t-stat	*P*-Value	FDR	Pearsons Correlation Coefficient	*P*-Value	FDR			
Age of Death	7	rs13479522	128,129,547	NM_021334	−0.30	−3.11	0.002	0.045	0.31	0.000	0.004	C57BL/6J	*Itgax*	*ITGAX*
	8	rs13479871	84,956,610	ENSMUST00000109736	0.08	3.57	0.001	0.014	−0.22	0.012	0.046	NOD/ShiLtJ	*Rnaseh2a*	*RNASEH2A*
DMFS	11	rs3711357	61,505,144	ENSMUST00000102657	0.16	6.64	9.15E-10	1.07E-07	-	0.004	0.021	NOD/ShiLtJ	*B9d1*	*B9D1*
Primary Tumor Burden	13	rs8267104	23,763,668	NM_030609	−0.42	−5.04	1.63E-06	9.88E-05	0.25	0.004	0.022	C57BL/6J	*Hist1h1a*	*HIST1H1A*
			23,751,088	NM_175660	−0.92	−10.75	2.50E-19	2.06E-16	0.24	0.008	0.034	C57BL/6J	*Hist1h2ab*	*HIST1H2AB*
			23,760,692	BC119241	−0.14	−3.50	0.001	0.017	0.32	0.000	0.003	C57BL/6J	*Hist1h4a*	*HIST1H4A*
			23,353,103	NM_001111107	−0.20	−5.84	4.42E-08	3.67E-06	0.29	0.001	0.006	NOD/ShiLtJ	*Zfp322a*	*ZNF322*
			23,535,418	ENSMUST00000080859	−0.20	−4.06	8.52E-05	0.003	0.22	0.012	0.046	C57BL/6J	*Hist1h3g*	*HIST1H3E*
			23,744,973	ENSMUST00000091752	−0.29	−6.45	2.39E-09	2.57E-07	0.25	0.004	0.020	C57BL/6J	*Hist1h3c*	*HIST1H3C*
			23,527,011	AK006302	−0.37	−6.87	2.88E-10	3.81E-08	0.28	0.002	0.011	C57BL/6J	*Hist1h4h*	*HIST1H4H*
		rs3720782	55,623,005	NM_007596	−0.10	−4.02	9.81E-05	0.004	0.24	0.006	0.029	C57BL/6J	*Caml*	*CAMLG*
			56,288,643	NM_019568	0.31	3.12	0.002	0.045	−0.36	3.73E-05	0.001	NOD/ShiLtJ	*Cxcl14*	*CXCL14*
		rs3679784	21,421,275	NM_001162920	0.21	3.58	4.81E-04	0.013	0.23	0.011	0.044	NOD/ShiLtJ	*Pgbd1*	*PGBD1*
		rs6275055	24,943,152	NM_008156	−0.51	−6.56	1.37E-09	1.52E-07	−0.32	0.000	0.002	C57BL/6J	*Gpld1*	*GPLD1*
Seminal Vesicle Tumor Burden	2	rs6209325	148,681,023	ENSMUST00000028928	0.09	3.40	0.001	0.022	−0.25	0.005	0.024	C57BL/6J	*Gzf1*	*GZF1*
		gnf02.149.271	151,494,182	NM_198326	−0.10	−4.93	2.58E-06	1.49E-04	−0.28	0.002	0.010	NOD/ShiLtJ	*Nsfl1c*	*NSFL1C*
		rs6247960	153,345,845	ENSMUST00000109790	−0.08	−3.22	0.002	0.034	−0.37	1.55E-05	0.000	C57BL/6J	*Asxl1*	*ASXL1*
		rs6376291	153,345,845		0.08	3.30	0.001	0.028	−0.37	1.55E-05	0.000	NOD/ShiLtJ	*Asxl1*	*ASXL1*
		rs13476860	155,817,730	NM_010808	0.16	6.17	9.02E-09	8.72E-07	−0.33	0.000	0.002	NOD/ShiLtJ	*Mmp24*	*MMP24*
	4	rs13477643	34,550,615	NM_001007589	0.13	4.44	1.94E-05	0.001	−0.33	0.000	0.002	NOD/ShiLtJ	*Akirin2*	*AKIRIN2*
			34,566,781	NM_015824	0.15	4.78	4.86E-06	2.65E-04	−0.47	2.38E-08	4.67E-06	NOD/ShiLtJ	*Orc3*	*ORC3*
		rs3698283	42,629,332	NM_011888	0.36	9.51	2.31E-16	9.27E-14	−0.41	1.71E-06	6.85E-05	NOD/ShiLtJ	*Ccl19*	*CCL19*
			42,916,660	ENSMUST00000107976	−0.23	−3.58	4.81E-04	0.014	−0.29	0.001	0.008	C57BL/6J	*N28178*	*KIAA1045*
			42,979,963	NM_009503	−0.14	−8.03	6.98E-13	1.55E-10	−0.24	0.006	0.029	C57BL/6J	*Vcp*	*VCP*
			42,714,926	NR_033123	−0.51	−5.55	1.70E-07	1.23E-05	−0.30	0.001	0.006	NOD/ShiLtJ	*4933409K07Rik*	None
			43,654,227	NM_026871	−0.15	−4.49	1.59E-05	7.52E-04	0.32	0.000	0.003	C57BL/6J	*Hint2*	*HINT2*
			42,736,593	ENSMUST00000144765	−0.37	−4.85	3.67E-06	2.05E-04	0.30	0.001	0.005	C57BL/6J	*Gm12395*	None
			42,206,998	ENSMUST00000169242	0.37	4.14	6.32E-05	0.002	−0.42	7.61E-07	3.69E-05	NOD/ShiLtJ	*Gm17167*	None
			42,244,362	BC059060	0.46	4.54	1.31E-05	0.001	−0.42	1.08E-06	5.03E-05	NOD/ShiLtJ	*Gm3893*	None
		rs13477643	34,949,074	NM_178061	−0.52	−5.95	2.58E-08	2.25E-06	0.28	0.001	0.009	C57BL/6J	*Mob3b*	*MOB3B*
			34,768,664	BC027508	−0.19	−7.35	2.48E-11	4.43E-09	0.31	0.000	0.004	C57BL/6J	*Smim8*	*SMIM8*
	8	rs13479922	92,855,350	NM_173014	−0.35	−3.83	1.98E-04	0.007	0.25	0.006	0.026	C57BL/6J	*Lpcat2*	*LPCAT2*
	17	rs3719497	24,528,251	NR_045289	0.19	4.35	2.79E-05	0.001	−0.30	0.001	0.006	NOD/ShiLtJ	*Rab26os*	None
		rs3719497	25,240,170	NM_026676	−0.10	−3.15	0.002	0.041	−0.23	0.011	0.043	C57BL/6J	*Tsr3*	*TSR3*
Liver Surface Metastasis Count	11	rs3023251	21,344,588	NR_035454	−0.48	−8.43	8.55E-14	2.23E-11	−0.25	0.005	0.023	C57BL/6J	*Mir1933*	None
														

### Expression Levels of QTL Candidate Genes Are Associated with Disease Free Survival in Two Human Prostate Tumor Datasets

Having used a highly stringent analytical approach to identify 35 aggressive tumorigenesis susceptibility genes in (TRAMP × NOD/ShiLtJ) F2 males, we aimed to determine whether the human orthologs of these genes play a similar role in human prostate cancer. Of the 35 QTL candidate genes identified in (TRAMP × NOD/ShiLtJ) F2 males, 29 had a human ortholog (Table 2). The 6 transcripts with no direct ortholog were omitted from further analyses owing to their probable irrelevance to human prostate cancer. We hypothesized that if the human orthologs of the remaining 29 QTL candidate genes play a similar role in aggressive prostate cancer susceptibility, they should exhibit the same characteristics that facilitated their identification in (TRAMP × NOD/ShiLtJ) F2 males. Specifically, their expression levels in primary tumors should be associated with aggressive prostate cancer, and they should be in linkage disequilibrium (LD) with germline SNPs associated with susceptibility to aggressive prostate cancer development.

To address the first of these, the expression levels of QTL candidate genes were examined in two publicly-accessible prostate cancer gene expression datasets using cBioPortal for Cancer Genomics (http://www.cbioportal.org/ [26], [27]), which is a web-based resource that comprises multi-dimensional cancer genomics data for numerous cancer subtypes. We initially focused on a prostate cancer dataset provided by The Cancer Genome Atlas (TCGA), which is comprised of a sufficient number of subjects to facilitate adequately powered survival analyses (TCGA [Provisional]). Here, cBioPortal reports static levels of gene expression in individual prostate tumors from this RNA-seq based dataset. Findings in TCGA (Provisional) cohort were confirmed in a second microarray-based dataset (Prostate Oncogenome Project [GSE21032]; [28]). Stepwise logistic regression analysis was performed to test the association between the expression levels of each of the 29 QTL candidate genes in the two datasets and dichotomized clinical variables, based on the common disease features reported for both cohorts (Figure S4). The comparisons of aggressive prostate cancer clinical variables used in logistic regression analyses, as well as the results of these tests are shown in Table 3. In TCGA (Provisional) cohort, the expression levels of three genes were positively correlated with aggressive disease characteristics. Specifically, the expression levels of *CXCL14* (odds ratio [OR]  = 1.62 [95% confidence interval 1.10–2.38]) and *RNASEH2A* (OR  = 2.17 [1.04–4.52]) were associated with disease recurrence; and *LPCAT2* (OR  = 1.44 [1.02–2.03]) with a higher pathological stage. In the GSE21032 cohort, the expression levels of three genes were associated with an increased risk of aggressive disease and two genes identified as having a protective effect. Specifically, the expression levels of *CXCL14* were associated with a higher pathological stage (OR  = 1.75 [1.19–2.59]). Divergent effects were observed for tumor Gleason score, with two genes being associated with a higher Gleason score (*ITGAX*; OR  = 3.87 [1.88–7.56] and *ZNF322*; OR  = 2.26 [1.27–4.02]) and two genes with a Gleason score <7 (*CCL19*; OR  = 0.46 [0.24–0.88] and *HIST1H1A*; (OR  = 0.45 [0.24–0.86]).

**Table 3:** Stepwise logistic regression analysis of QTL candidate genes in TCGA (Provisional) and GSE21032 cohorts.

Cohort	Clinical Trait	Clinical Trait Comparison	Gene	Odds Ratio	95% CI	*P*-Value
TCGA (Provisional)	Disease Free Status	Disease free vs. recurred	***CXCL14***	1.62	1.10–2.38	0.014
			***RNASEH2A***	2.17	1.04–4.52	0.038
	Pathological Stage	T2 vs. T3+T4	***LPCAT2***	1.44	1.02–2.03	0.038
GSE21032	Pathological Stage	T2 vs. T3+T4	***CXCL14***	1.75	1.19–2.59	0.005
	Gleason Score	<7 vs.≥7	***CCL19***	0.46	0.24–0.88	0.019
			***HIST1H1A***	0.45	0.24–0.86	0.017
			***ITGAX***	3.78	1.88–7.56	2.00E-04
			***ZNF322***	2.26	1.27–4.02	0.006

**Figure 3:**
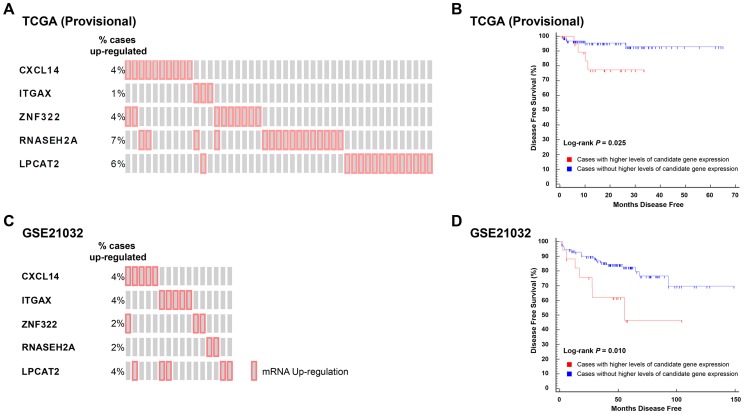
Higher levels of five QTL candidate genes are associated with poor DFS in TCGA (Provisional) and GSE21032 prostate cancer gene expression datasets. (A) ‘Oncoprint’ analysis demonstrates that 45/246 (18%) of cases in TCGA (Provisional) gene expression dataset have exclusively higher than average expression levels of five QTL candidate genes. (B) These higher levels of expression are associated with a reduced DFS in TCGA (Provisional) cohort. (C). Oncoprint' analysis demonstrates that 16/131 (12%) of cases in the GSE21032 gene expression dataset have exclusively higher than average expression levels of the same five QTL candidate genes. (D) As was the case with TCGA (Provisional) dataset, higher levels of expression of these genes is associated with a reduced DFS in the GSE21032 cohort.

To test the correlation between candidate gene expression and disease recurrence, genes implicated in aggressive disease development in logistic regression analyses performed in TCGA (Provisional) and GSE21032 cohorts were combined to create two gene sets: 1) a set of five genes associated with an increased propensity for aggressive disease development (*CXCL14*, *ITGAX*, *LPCAT2*, *RNASEH2A*, and *ZNF322*); and 2) a set of two genes with a protective effect (*CCL19* and *HIST1H1A*). The expression levels of transcripts within these two gene sets were correlated with disease free survival (DFS) using Kaplan-Meyer survival analysis in the TCGA (Provisional) cohort. Specifically, DFS was compared between cases with higher or lower levels of expression of one or more gene in either of the two gene sets to those cases with normal levels of expression of the same genes. Eighteen percent (45/246) of cases in TCGA (Provisional) dataset exhibited divergent levels of one or more of the five genes positively correlated with aggressive disease development (Figure 3A). Strikingly, the directionality of expression was significantly higher than average for each of the five genes in all 45 cases. Accordingly, Kaplan-Meyer survival analysis demonstrated that higher than average expression levels of one or more of these genes was associated with a poorer DFS (log-rank *P* = 0.025; Figure 3B). To confirm the findings from this cohort, survival analysis was performed in the GSE21032 dataset by comparing DFS in patients with higher than average levels of the five candidate genes compared to all other cases. In the GSE21032 dataset, 12% of cases (16/131) exhibited exclusively higher than average levels of expression of one or more candidate genes (Figure 3C). As was the case in TCGA (Provisional) dataset, higher than average levels of expression of these genes was associated with a poorer DFS (log-rank *P* = 0.010; Figure 3D).

Analysis of both datasets in cBioPortal showed that none of the cases with higher levels of candidate gene expression in either cohort exhibited either copy number alteration or somatic mutations of these genes. This implies that candidate gene copy number alteration and/or somatic mutations likely have no influence upon DFS in these datasets. Similar analysis in larger prostate cancer datasets will be required to confirm or refute whether the observed associations in the TCGA (Provisional) and GSE21032 are correlated with primary tumor copy number variation. Finally, the expression levels of the two genes that were negatively correlated with aggressive disease on logistic regression were not correlated with DFS in either cohort.

### Analysis of Human Prostate Cancer GWAS Data Reveals That QTL Candidate Gene SNPs Are Associated with Aggressive Prostate Cancer

Our QTL mapping strategy demonstrates that QTL candidate gene germline variation is associated with aggressive tumorigenesis in the TRAMP mouse. To evaluate whether this is the case for the human orthologs of these genes, SNP allele frequencies were evaluated in a publicly available human prostate cancer GWAS dataset. Specifically, these analyses were performed using the Cancer Genetic Markers of Susceptibility (CGEMS) GWAS, which consists of 1,172 prostate cancer patients and 1,157 controls of European ancestry from the Prostate, Lung, Colon and Ovarian (PLCO) Cancer Screening Trial [29], [30]. This relatively well-studied resource has facilitated the identification of novel loci associated with prostate cancer, including a second prostate cancer risk locus at 8q24 [31].

Given that we hypothesized that QTL candidates modulate prostate cancer aggressiveness but not prostate cancer initiation, controls were omitted from analyses. The CGEMS cohort is well suited for this purpose, with the case cohort subdivided into non-aggressive (Gleason score <7 and stage <III; n = 484) and aggressive (Gleason score ≥7 or stage ≥III; n = 688) cases. In addition to these clinical characteristics, case-case analyses were performed for the additional aggressive disease variables shown in Table 4. These variables related to the size or direct extent of the primary tumor (pros_stage_t), local metastasis to lymph nodes (pros_stage_n) and distant metastasis (pros_stage_m). We elected to include these variables to more closely reflect the phenotypes used to identify QTL candidate genes in (TRAMP × NOD/ShiLtJ) F2 mice.

**Table 4:** Clinical variables analyzed in CGEMS GWAS.

PLCO Variable	Description	GWAS Comparison Performed
pros_stage	Prostate Cancer Stage	Stage I+II vs. stage III+IV
pros_stage_t	T Stage Component (Primary Tumor)	T1+T2 vs. T3+T4
pros_stage_n	N Stage Component (Nodal Involvement)	N0 vs. N1+N2
pros_stage_m	M Stage Component (Distant Metastases)	M0 vs. M1A+M1B+M1C
pros_gleason	Best Gleason Score Available	Gleason score <7 vs.≥7
pros_gleason_biop	Biopsy Gleason Score	Gleason score <7 vs.≥7
pros_gleason_prost	Prostatectomy Gleason Score	Gleason score <7 vs.≥7

In the study, 1,317 SNPs were mapped within a 100 kb radius of the 29 QTL candidate genes were tested in the CGEMS cohort. Analysis of aggressive vs. non-aggressive disease phenotypes were performed as per the comparisons described in Table 4. Correction for multiple testing was performed using permutation testing (n = 10,000 permutations). Fourteen of the 29 candidate genes exhibited evidence for association with clinical characteristics of aggressive prostate cancer (Table 5). Most notably, SNPs in three of the five genes associated with poor clinical outcomes in TCGA (Provisional) and GSE21032 prostate cancer gene expression datasets (*CXCL14*, *ITGAX*, and *LPCAT2*) were all associated with aggressive prostate cancer: for *CXCL14*, associations were evident between rs801564 and metastasis to regional lymph nodes (permutation *P* = 0.011; OR = 1.05 [1.01–1.09]), and between rs10515473 and Gleason score at prostatectomy (permutation *P* = 0.001; OR = 0.72 [0.59–0.88]); for *ITGAX*, an association was apparent between rs8047538 and Gleason score at prostatectomy (permutation *P* = 0.007; OR  = 1.33 [1.08–1.62]); and for *LPCAT2*, associations were evident between rs3764263 and primary tumor stage (permutation *P* = 0.009; OR = 1.61 [1.12–2.31]), between rs289707 and biopsy Gleason score (permutation *P* = 0.002; OR = 1.22 [1.07–1.38]), between rs2289119 and metastasis to regional lymph nodes (permutation *P* = 0.009; OR = 1.06 [1.01–1.10]), and between rs17369578 and best Gleason score available (permutation *P* = 0.003; OR = 1.41 [1.13–1.77]). Manhattan plots for all relevant genomic regions are shown in Figure S5. Additionally, rare haplotypes (<1% frequency) in LD with three QTL candidate genes were associated with clinical markers of prostate cancer aggressiveness (Table S15).

**Table 5:** QTL candidate gene SNPs associated with aggressive prostate cancer in CGEMS GWAS.

Chr.	Candidate Gene	SNP Distance From Gene (bp)	SNP	PLCO Variable	Odds Ratio (95% C.I.)	Minor Allele Frequency		t stat	*P* value	Permutation *P* value
						Aggressive Disease	Non-Aggressive Disease			
5q31.1	***CXCL14***	97285	rs801564	pros_stage_n	1.05 (1.01–1.09)	0.001	0.282	2.587	0.010	0.011
		47017	rs10515473	pros_gleason_prost	0.72 (0.59–0.88)	0.163	0.122	−3.148	0.002	0.001
6p22.1	*HIST1H3E*	6834	rs933199	pros_gleason	0.75 (0.62–0.92)	0.032	0.025	−2.759	0.006	0.006
		17168	rs198806	pros_gleason_prost	0.77 (0.63–0.93)	0.218	0.172	−2.643	0.008	0.009
	*PGBD1*	31884	rs1233708	pros_stage	1.08 (1.02–1.13)	0.042	0.213	2.921	0.004	0.004
6p22.2	*HIST1H1A*	13705	rs6910741	pros_stage_n	0.94 (0.90–0.98)	0.002	0.144	−2.620	0.009	0.009
6p22.3	*GPLD1*	0	rs793663	pros_gleason_prost	1.32 (1.09–1.60)	0.186	0.195	2.854	0.004	0.004
		37981	rs3789224	pros_stage_m	1.08 (1.02–1.13)	4.06E-04	0.127	2.627	0.009	0.010
6q15	*AKIRIN2*	0	rs7755167	pros_gleason	0.86 (0.78–0.95)	0.246	0.221	−3.107	0.002	0.002
	*ORC3*	40224	rs9450716	pros_gleason	1.16 (1.05–1.28)	0.161	0.189	3.042	0.002	0.002
9p13.3	*CCL19*	80925	rs3802427	pros_stage_m	1.09 (1.04–1.15)	4.06E-04	0.162	3.557	3.89E-04	3.00E-04
	*KIAA1045*	78052	rs10123308	pros_gleason_prost	1.30 (1.08–1.57)	0.212	0.216	2.773	0.006	0.005
9p21.2	*MOB3B*	3431	rs3849942	pros_stage_t	2.15 (1.41–3.28)	0.161	0.075	3.564	3.80E-04	5.00E-04
		0	rs3739530	pros_gleason	0.80 (0.70–0.93)	0.075	0.056	−2.996	0.003	0.002
		74176	rs1853186	pros_stage	0.94 (0.90–0.98)	0.079	0.272	−2.730	0.006	0.006
		0	rs10121765	pros_gleason_prost	0.77 (0.64–0.93)	0.260	0.209	−2.776	0.006	0.005
16p11.2	***ITGAX***	9009	rs8045738	pros_gleason_prost	1.33 (1.08–1.62)	0.141	0.148	2.753	0.006	0.007
16q12.2	***LPCAT2***	19704	rs3764263	pros_stage_t	1.61 (1.12–2.31)	0.324	0.134	2.591	0.010	0.009
		0	rs289707	pros_gleason_biop	1.22 (1.07–1.38)	0.075	0.126	3.059	0.002	0.002
		72508	rs2289119	pros_stage_n	1.06 (1.01–1.10)	0.001	0.187	2.591	0.010	0.009
		0	rs17369578	pros_gleason	1.41 (1.13–1.77)	0.020	0.026	2.976	0.003	0.003
20p11.21	*GZF1*	836	rs6076072	pros_stage_t	2.17 (1.21–3.92)	0.068	0.035	2.589	0.010	0.008
20p13	*NSFL1C*	4463	rs6042568	pros_gleason	0.75 (0.62–0.90)	0.037	0.029	−3.007	0.003	0.003

QTL candidate genes also identified as having a dysregulated expression in prostate cancer gene expression datasets are denoted in bold typeface.

## Discussion

A systems genetics approach has been employed in this study to identify three novel susceptibility genes for aggressive prostate cancer, and to the best of our knowledge, this is the first study of its type to use this approach in this form of cancer. The three high priority candidate genes identified in QTL mapping studies using the TRAMP mouse model have diverse cellular functions (Table 6), and have not been previously implicated as germline susceptibility genes for aggressive prostate cancer. Functional characterization of these genes to clarify their role in aggressive prostate cancer is therefore of much importance. However, given the strength of the genetic and genomic data implicating each of these genes in aggressive tumorigenesis, we argue that the required depth of such functional characterization is beyond the scope of the current study. Nevertheless, other studies support the role of some of these genes in aggressive tumorigenesis. For example, higher levels of expression of *LPCAT2* are observed in a diverse range of tumors, notably breast and cervical carcinomas [32]. Additionally, a linkage study demonstrated that *CXCL14* resides in a risk locus for aggressive prostate cancer in the 5q31 region [33], and higher levels of this gene have been observed in tumors with a higher Gleason score [34]. Concomitantly, over-expression of *CXCL14* in fibroblasts stimulates tumor angiogenesis and growth of prostate cancer cells [35] through activation of NOS1-derived nitric oxide signaling pathways [36]. These findings are in keeping with the results of our survival analyses of TCGA (Provisional) and the GSE21032 cohorts, which demonstrated that higher than average levels of expression of *CXCL14* in bulk tumor tissue is associated with an increased risk of recurrence.

**Table 6:** High priority aggressive prostate cancer susceptibility genes and associated aggressive disease traits form each element of this study.

Aggressive Prostate Cancer Susceptibility Gene	Name	Cellular Function	OMIM	Associated Aggressive Disease Traits		
				(TRAMP × NOD/ShiLtJ) F2 QTL Analysis	Logistic Regression in Human Gene Expression Datasets	CGEMS GWAS Associations
*CXCL14*	*Chemokine (C-X-C motif) ligand 14*	Homeostasis of monocyte-derived macrophages	604186	Primary tumor burden	Disease free status; pathological stage	Nodal metastasis; Gleason score at prostatectomy
*ITGAX*	*Integrin, Alpha × (complement component 3 receptor 4 subunit)*	Cell-cell adhesion	151510	Age of death	Gleason score	Gleason score at prostatectomy
*LPCAT2*	*Lysophosphatidylcholine acyltransferase 2*	Membrane biogenesis; production of platelet-activating factor in inflammatory cells	612040	Seminal vesicle tumor burden	Pathological stage	Nodal metastasis; biopsy Gleason score; best Gleason score; pathological stage

Identification of these novel aggressive prostate cancer susceptibility genes has been facilitated through use of the TRAMP mouse model. However, the NE histological phenotype of tumors and the use of the non-physiological SV40 T-antigen to induce tumorigenesis have led to criticism of TRAMP [37]. The validity of these criticisms is, however, being increasingly questioned, particularly in light of the probable increase in incidence of human NE prostate tumors induced by increasingly efficacious ADTs [38]. The TRAMP model can therefore be viewed as a powerful tool to study the pathogenesis of NE forms of aggressive, castrate-resistant disease. Additionally, the SV40 T-antigen directly inactivates Rb and p53 [39], and the aggressive disease seen in TRAMP mice therefore mimics somatic mutation of these potent tumor suppressors. We do, however, acknowledge that observations from the TRAMP model are sometimes not directly comparable to human prostate cancer. An example from the current study would be the association of higher levels of *Cxcl14*/*CXCL14* being negatively associated with primary tumor burden in (TRAMP × NOD/ShiLtJ) F2 mice but positively correlated with disease recurrence in humans. Additionally, the traits used to nominate candidate genes in (TRAMP × NOD/ShiLtJ) F2 mice frequently differ from the associated aggressive disease traits observed in human populations, as illustrated in Table 6. We therefore regard the TRAMP model as a powerful tool for nominating aggressive disease modifiers in a generalized sense, and the integration of different lines of evidence from human prostate cancer populations is of critical importance for deciphering the relevance of observations derived from mice.

The integration of these different lines of evidence from human prostate cancer datasets to validate findings from our genetic screen in the TRAMP mouse has proven a pivotal element of this study. There are, however, a number of aspects of our analysis of the CGEMS GWAS data that warrant further discussion. First, we acknowledge that our use of a permutation test does not fully resolve the issue of correcting for type I errors. Rather, permutation testing has allowed us to report *P*-values that are both more stable and accurate than uncorrected values. Second, we also recognize that a genome-wide level of significance was not achieved with any of the SNPs characterized in the CGEMS GWAS dataset. One probable reason for this is the limited statistical power of the case-case analysis performed here, which reflects the relatively small study population. Validation of these findings in additional prostate cancer cohorts is therefore vital. However, this lack of genome-wide significance may reflect one of the few limitations of GWAS. Specifically, although GWAS have revolutionized our understanding of complex trait susceptibility, they have not yet been able to explain the complete influence of heritability on disease susceptibility. This is true of prostate cancer, where all of the variants thus far identified by GWAS are estimated to explain less than one third of familial disease risk [11], [40]. It has been postulated that a possible reason for this is that biologically relevant modifiers that achieve the *P*<0.05 nominal level of significance are being missed since they do not reach the necessarily stringent level of genome-wide significance [41]. Therefore, alternative methodologies to augment GWAS, including the types of approaches described here, may facilitate characterization of some of this ‘missing heritability’. Thus, the evidence for association between QTL candidate gene SNPs and aggressive disease development from these GWAS data in this study is insufficient in isolation. However, the power of these GWAS analyses is derived from consideration in unison with the mouse and human gene expression data.

In summary, we have identified *CXCL14, ITGAX* and, *LPCAT2* as novel susceptibility genes for aggressive prostate cancer development. This is the first study of its type to address the influence of germline polymorphism on tumor progression and metastasis in prostate cancer using systems genetics approach. Additionally, this approach has identified novel modifiers of aggressive prostate cancer that might not be readily apparent through human association studies. Knowledge of these variants will allow for more accurate determination of a patient's risk of metastasis, thus improving prognostic accuracy and facilitating more personalized treatments.

## Methods

### Animal Husbandry and Genotyping

C57BL/6J-Tg(TRAMP)824Ng/J (TRAMP) and NOD/ShiLtJ mice were obtained from The Jackson Laboratory (Bar Harbor, ME). F1 mice were generated by crossing TRAMP females, which were hemizygous for PB-TAg transgene (Tg), to NOD/ShiLtJ males. F2 mice were generated by crossing Tg+ F1 females with Tg- F1 males. All animals were handled, housed and used in the experiments humanely in accordance with the NHGRI Animal Care and Use Committee guidelines. All work was performed under Animal Study Protocol G-09-2. Mouse tail genomic DNA was extracted from F1 progeny with the HotSHOT method [42] for genotyping analysis. PCR screening was performed as described [14] to identify the hemizygous PB-TAg transgene positive F1 and F2 mice.

### Tissue Collection

As described previously in [15], (TRAMP × NOD/ShiLtJ) F2 male mice were sacrificed by pentobarbital overdose at 30 weeks of age or humane endpoint, whichever was achieved first. Humane experimental endpoints for this study were rapid weight loss, hunched posture, labored breathing, trauma, impaired mobility, dysuria, or difficulty in obtaining food or water. Prostate tumor, seminal vesicles, lungs, liver, and lymph nodes were harvested from (TRAMP × NOD/ShiLtJ) F2 males. Prostate tumor and seminal vesicles were weighed to quantify tumor burden. Visible, enlarged lymph nodes in para-aortic region were weighed to quantify metastatic lymph node burden. Lungs were collected to determine isolated tumor cell infiltrates in lung parenchyma and microscopic metastatic lesions. Other organs displaying macroscopic metastatic lesions through gross observation were also collected for histology. These collected tissues were fixed in buffered formalin (10% w/v phosphate buffered formaldehyde, Fisher Scientific) overnight and then transferred to 70% ethanol. Fixed tissues were embedded in paraffin, sectioned to a thickness of 4 µm and stained with hematoxylin and eosin (H&E). Histology slides were scanned with Scanscope Digital microscope (Aperio, Vista, CA).

### SNP Genotyping

Genomic DNA was extracted from F2 tail biopsies using a Gentra Puregene DNA Extraction Kit (Qiagen, Valencia, CA), per the manufacturers protocol. Five microliters of DNA at 75 ng/µl was used for SNP genotyping using the 1536 plex assay kit and GoldenGate Assay Mouse Medium Density Linkage Array following the manufacturers protocol (Illumina, San Diego, CA). The intensity data for each SNP for 228 samples were normalized and the genotypes assigned using Illumina GenomeStudio Genotyping Analysis Module version 1.9.4. SNPs with a GC score <0.7 and non-informative (homozygous) SNPs were excluded from further analysis. SNP Hardy–Weinberg equilibrium (HWE) P-values were estimated with PLINK. SNPs were omitted if the HWE *P*<0.001.

### Microarray Analysis

As described previously in [43], total RNA extractions from (TRAMP × NOD/ShiLtJ) F2 tumor samples were carried out using TRIzol Reagent (Life Technologies, Inc.) according to the standard protocol. RNA quality and quantity was ensured using the Bioanalyzer (Agilent, Inc., Santa Clara, CA) and NanoDrop (Thermo Scientific, Inc., Waltham, MA), respectively. Per RNA labeling, 200 ng of total RNA was used in conjunction with the Affymetrix (Santa Clara, CA) recommended protocol for the GeneChip 2.0 ST chips. Hybridization cocktails containing the fragmented and labeled cDNAs were hybridized to Affymetrix Mouse Genome 2.0 ST GeneChip. Chips were washed and stained by the Affymetrix Fluidics Station using the standard format and protocols as described by Affymetrix. Probe arrays were stained with streptavidin phycoerythrin solution (Molecular Probes, Carlsbad, CA) and enhanced by using an antibody solution containing 0.5 mg/mL of biotinylated anti-streptavidin (Vector Laboratories, Burlingame, CA). An Affymetrix Gene Chip Scanner 3000 was used to scan the probe arrays. Gene expression intensities were calculated using Affymetrix AGCC software. Partek Genomic Suite was used to RMA normalize (Robust Multichip Analysis), summarize, log2 transform the data, run ANOVA analysis and unsupervised hierarchical clustering. To account for genes expressed below the threshold of detection, average levels of gene expression across all samples were calculated and genes expressed in the lower 10^th^ percentile excluded. This encompassed the average experiment-wide background intensity of 3.04±0.12.

### Accession Numbers

Microarray data are available through Gene Expression Omnibus (accession no. GSE58829).

### Statistical Analysis of Data from (TRAMP × NOD/ShiLtJ) F2 Mice

QTL analysis was performed using J/qtl [20]. Mapping of QTLs was performed for all traits using a single-QTL analysis, using a binary model for binary traits (e.g., distant metastasis free survival [DMFS]) and a non-parametric model for all other traits. Significance levels were computed using permutation testing [44], using 10,000 permutations. Age of death was used as an additive covariate for tumor-related traits (primary tumor burden, seminal vesicle tumor burden). Age and primary tumor burden were used as additive covariates for all metastasis-related traits. Confidence intervals for QTLs identified were estimated using 2-LOD support intervals, which is on the chromosome where the LOD score did not fall below 2.0 of its maximum [45]. Only those QTLs reaching a genome-wide α<0.05 were considered to be of interest.

eQTL analysis was performed using Matrix-eQTL in R [24]. A linear model was used to test for association between gene expression and SNPs, with age and primary tumor burden used as covariates. A SNP that mapped ≤1 Mb upstream or downstream of the transcription start site was used to define proximal eQTLs. Correction for multiple testing was performed using the Benjamini-Hochberg FDR method. An FDR <0.05 was used as the threshold for significant eQTLs.

Pearson correlation coefficients and associated *P*-values were calculated for all traits other than those with a binary distribution by correlating the log2 transformed expression intensities of all probes mapped to a given QTL with the relevant QTL trait using MedCalc (Ostend, Belgium). For the latter, student's t-tests were performed to test the significance of transcript-trait correlations. Correction for multiple testing was performed using the Benjamini-Hochberg FDR method using the QVALUE module in R [46]. An FDR <0.05 was used as the threshold for significant correlations.

### Analysis of Human Prostate Cancer Gene Expression Datasets

QTL candidate gene expression levels were analyzed in the cBioPortal for Cancer Genomics database (http://www.cbioportal.org; [27]). Two human prostate cancer datasets possessed sufficient gene expression and clinical data to facilitate assessment of candidate genes: a) TCGA (Provisional) – the Cancer Genome Atlas provisional data (https://tcga-data.nci.nih.gov/tcga/tcgaCancerDetails.jsp?diseaseType=PRAD&diseaseName=Prostate%20adenocarcinoma); and b) GSE21032 - Prostate Oncogenome Project, Taylor et al. [28]. The gene expression levels in TCGA (Provisional) dataset available on the cBioPortal website are provided by The Cancer Genome Atlas. Here, level 3 expression data were generated from RNA-seq data by first generating ‘Reads per Kilobase per Million mapped reads’ (RPKM; [47]) counts. This is followed by utilization of MapSplice [48] to align sequence reads and ‘RNA-Seq by Expectation Maximization’ (RSEM) values [49] to perform gene quantitation. cBioPortal reports higher or lower levels of gene expression by a z-score of ≥2 or ≤−2, respectively, where the z-score is the standard deviation of static levels of transcript expression in a given case compared to the mean transcript expression in diploid tumors. Diploid tumors were used for the purposes of normalization since candidate gene ploidy could presumably impact average expression levels of candidate genes.

In the GSE21032 cohort, gene up- or down-regulation in a given case is again provided by cBioPortal as a z-score of ≥2 or ≤−2, respectively. However, here a z-score of 2 was defined as an array probe-set intensity that is two standard deviations greater than the mean of the probe set intensity in the matched normal tissue, with the opposite being true for down-regulated genes. Therefore, to make candidate gene expression levels more comparable to those reported for TCGA (Provisional) cohort, raw gene expression data for GSE21032 were downloaded from cBioPortal (http://cbio.mskcc.org/cancergenomics/prostate/data/MSKCC_PCa_mRNA_data.zip). The expression levels of the 29 QTL candidate genes were subsequently extracted of all primary tumors with mRNA data (n = 131), average expression levels and standard deviations calculated, and z-scores for candidate gene expression in individual tumors calculated using the following formula: ([gene expression in individual tumor – average population gene expression]/population expression standard deviation).

Logistic regression and Kaplan-Meyer survival analyses were performed using MedCalc (Ostend, Belgium). Logistic regression was performed using the stepwise method, with individual dichotomized clinical variables (Table 3; Figure S4) as dependent variables and z-scores for all 29 candidate genes as independent variables. Kaplan-Meyer survival curves were constructed by comparing the time to recurrence in cases from either cohort with higher levels of tumor candidate gene expression *versus* all other cases.

### Statistical Analysis of CGEMS GWAS

The clinical characteristics of the CGEMS GWAS cohort have been described extensively elsewhere (dbGaP Study Accession: phs000207.v1.p1; [31]). All SNPs analyzed were either located within a given QTL candidate gene or no more than 100,000 bp upstream or downstream. SNP HWE *P*-values were estimated with PLINK. SNPs were omitted if the HWE *P*<0.001. Association analysis between aggressive prostate cancer phenotype and SNP or haplotype was performed using a generalized linear model (glm). Age and PC1, PC2 and PC3 were included as covariates in the glm. Analysis of aggressive vs. non-aggressive disease phenotypes were performed as per the comparisons described in Table 3. Correction for multiple testing was performed using permutation testing (n = 10,000 permutations) using the glm on NIH biowulf super cluster computer system (http://biowulf.nih.gov). Specifically, permutation testing was performed for each phenotype against one SNP under rearrangements of the labels on all individuals with 10,000 times. Permutation tests were performed only in instances where the uncorrected *P*<0.01. Manhattan plots were constructed in R. For haplotype analysis, genome-wide LD blocks were estimated by using the Solid Spine algorithm of Haploview software with the default parameters, and fastPHASE was performed to generate haplotypes for each individual based on the LD blocks on NIH biowulf super cluster computer system (http://biowulf.nih.gov). FDR *P*-values were calculated by the MULTITEST package of R. All analyses were performed by using R.

## Supporting Information

Figure S1 Correlations between tumor- and metastasis-related traits in (TRAMP × NOD/ShiLtJ) F2 mice. Primary prostate tumor burden exhibited a negative correlation with age of death (A) and positive correlations with DMFS (B), lymph node metastasis (C), and lymph node metastasis burden (D). Conversely, seminal vesicle tumor burden was positively correlated with age of death (E) and negatively correlated with DMFS (F), lymph node metastasis (G), and lymph node metastasis burden (H).
(TIF)

Figure S2 Correlation between seminal vesicle tumor burden and primary tumor burden in (TRAMP × NOD/ShiLtJ) F2 mice.
(TIF)

Figure S3 QTL plots for aggressive disease loci identified in (TRAMP × NOD/ShiLtJ) F2 mice. QTLs were observed for the following traits: (A) DMFS; (B) total nodal metastasis burden; (C) liver surface metastasis count; (D) prostate tumor burden; (E) seminal vesicle tumor burden; and (F) age of death. The horizontal dotted line represents a genome-wide level of statistical significance of α <0.05.
(TIF)

Figure S4 Clinical characteristics of patients represented in the GSE21032 and TCGA (Provisional) datasets.
(TIF)

Figure S5 Manhattan plots for genomic regions of interest in CGEMS GWAS. Plots are only shown for regions where candidate gene SNPs were associated with the following phenotypes: (A) best Gleason score available; (B) biopsy Gleason score; (C) prostatectomy Gleason score; (D) prostate cancer stage; (E) distant metastasis; (F) nodal involvement; and (G) primary tumor stage.
(TIF)

Table S1 Distribution of aggressive prostate cancer phenotypes across (TRAMP × NOD/ShiLtJ) F2 mice.
(XLSX)

Table S2 Proximal eQTLs in (TRAMP × NOD/ShiLtJ) F2 mice.
(XLSX)

Table S3 Distal and *trans-*eQTLs in (TRAMP × NOD/ShiLtJ) F2 mice.
(XLSX)

Table S4 Correlation analysis for the microarray expression level of transcripts located within the chromosome 1 DMFS QTL with DMFS.
(XLSX)

Table S5 Correlation analysis for the microarray expression level of transcripts located within the chromosome 11 DMFS QTL with DMFS.
(XLSX)

Table S6 Correlation analysis for the microarray expression level of transcripts located within the chromosome 13 lymph node metastasis burden QTL with lymph node metastasis burden.
(XLSX)

Table S7 Correlation analysis for the microarray expression level of transcripts located within the chromosome 11 liver surface metastasis count QTL with liver surface metastasis count.
(XLSX)

Table S8 Correlation analysis for the microarray expression level of transcripts located within the chromosome 13 primary tumor burden QTL with primary tumor burden.
(XLSX)

Table S9 Correlation analysis for the microarray expression level of transcripts located within the chromosome 2 seminal vesicle tumor burden QTL with seminal vesicle tumor burden.
(XLSX)

Table S10 Correlation analysis for the microarray expression level of transcripts located within the chromosome 4 seminal vesicle tumor burden QTL with seminal vesicle tumor burden.
(XLSX)

Table S11 Correlation analysis for the microarray expression level of transcripts located within the chromosome 8 seminal vesicle tumor burden QTL with seminal vesicle tumor burden.
(XLSX)

Table S12 Correlation analysis for the microarray expression level of transcripts located within the chromosome 17 seminal vesicle tumor burden QTL with seminal vesicle tumor burden.
(XLSX)

Table S13 Correlation analysis for the microarray expression level of transcripts located within the chromosome 7 age of death QTL with age of death.
(XLSX)

Table S14 Correlation analysis for the microarray expression level of transcripts located within the chromosome 8 age of death QTL with age of death.
(XLSX)

Table S15 Statistically significant aggressive disease-associated haplotypes for QTL candidate genes in the CGEMS prostate cancer cohort.
(XLSX)
