# Correction: A Systems Genetics Approach Identifies *CXCL14*, *ITGAX*, and *LPCAT2* as Novel Aggressive Prostate Cancer Susceptibility Genes

**DOI:** 10.1371/journal.pgen.1005127

**Published:** 2015-05-01

**Authors:** The PLOS Genetics Staff

The authors made an error in analysis, with SNPs being mapped to human genome build hg18 and genes to human genome build hg19. Consequently, there are errors in Table 5, S15 Table, and the accompanying text on pages 9 and 11. Corrected text and tables are provided here.

**Table 5:** QTL candidate gene SNPs associated with aggressive prostate cancer in CGEMS GWAS.

Chr.	Candidate Gene	SNP Distance From Gene (bp)	SNP	PLCO Variable	Odds Ratio (95% C.I.)	Minor Allele Frequency		t stat	*P* value	Permutation *P* value
						Aggressive Disease	Non-Aggressive Disease			
5q31.1	***CXCL14***	0	rs2547	pros_gleason_prost	0.61 (0.43-0.85)	0.054	0.033	-2.889	0.004	0.003
		0	rs2237061	pros_gleason_prost	0.61 (0.43-0.88)	0.048	0.030	-2.685	0.007	0.008
		19118	rs10515473	pros_gleason_prost	0.72 (0.59-0.88)	0.163	0.122	-3.148	0.002	0.002
		24155	rs4463175	pros_gleason_prost	1.42 (1.12-1.81)	0.087	0.101	2.878	0.004	0.003
		69386	rs801564	pros_stage_n	1.05 (1.01-1.09)	0.001	0.282	2.587	0.010	0.010
		92585	rs2067000	pros_stage_n	1.05 (1.02-1.09)	0.001	0.288	2.883	0.004	0.004
6p22.1	*PGBD1*	76094	rs1233708	pros_stage	1.08 (1.02-1.13)	0.042	0.213	2.921	0.004	0.003
6p22.2	*HIST1H2AB*	59345	rs1800562	pros_gleason	1.40 (1.16-1.70)	0.025	0.041	3.459	0.001	0.001
		79097	rs933199	pros_gleason	0.75 (0.62-0.92)	0.032	0.025	-2.759	0.006	0.007
6p22.3	*GPLD1*	43164	rs811103	pros_gleason_prost	1.32 (1.09-1.60)	0.186	0.195	2.855	0.004	0.005
		56546	rs793663	pros_gleason_prost	1.32 (1.09-1.60)	0.186	0.195	2.854	0.004	0.006
6q15	*AKIRIN2*	0	rs4707385	pros_gleason	1.16 (1.05-1.29)	0.135	0.164	2.909	0.004	0.003
	*ORC3*	0	rs6930600	pros_gleason	0.86 (0.78-0.95)	0.246	0.222	-3.095	0.002	0.002
		0	rs7755167	pros_gleason	0.86 (0.78-0.95)	0.246	0.221	-3.107	0.002	0.002
		0	rs7772351	pros_gleason	1.14 (1.04-1.26)	0.164	0.193	2.768	0.006	0.007
		0	rs9450765	pros_gleason	1.17 (1.06-1.29)	0.171	0.202	3.118	0.002	0.002
		43692	rs7757636	pros_gleason	0.86 (0.79-0.95)	0.246	0.222	-3.089	0.002	0.003
		96943	rs9450716	pros_gleason	1.16 (1.05-1.28)	0.161	0.189	3.042	0.002	0.002
9p13.3	*CCL19*	70925	rs3802427	pros_stage_m	1.09 (1.04-1.15)	0.000	0.162	3.557	3.89E-04	1.00E-04
	*CCL21*	75161	rs277606	pros_gleason_prost	1.35 (1.11-1.65)	0.179	0.187	2.986	0.003	0.002
	*KIAA1045*	68052	rs10123308	pros_gleason_prost	1.30 (1.08-1.57)	0.212	0.216	2.773	0.006	0.006
9p21.2	*MOB3B*	0	rs868856	pros_stage	0.94 (0.89-0.98)	0.064	0.215	-2.748	0.006	0.006
		0	rs868856	pros_stage_t	2.02 (1.34-3.04)	0.191	0.088	3.371	0.001	0.001
		0	rs3739530	pros_gleason	0.80 (0.70-0.93)	0.075	0.056	-2.996	0.003	0.004
		0	rs4879515	pros_stage_t	1.68 (1.16-2.41)	0.320	0.130	2.784	0.005	0.005
		0	rs7046653	pros_stage_t	2.01 (1.34-3.02)	0.192	0.088	3.346	0.001	0.001
		0	rs7046653	pros_stage	0.94 (0.89-0.98)	0.064	0.216	-2.707	0.007	0.007
		0	rs10121765	pros_gleason_prost	0.77 (0.64-0.93)	0.260	0.209	-2.776	0.006	0.005
		6547	rs2814707	pros_stage_t	2.09 (1.38-3.19)	0.162	0.075	3.458	0.001	0.001
		13431	rs3849942	pros_stage_t	2.15 (1.41-3.28)	0.161	0.075	3.564	3.80E-04	0.001
		31199	rs774359	pros_stage_t	1.99 (1.32-3.01)	0.176	0.079	3.284	0.001	0.001
		64176	rs1853186	pros_stage	0.94 (0.90-0.98)	0.079	0.272	-2.730	0.006	0.006
16p11.2	***ITGAX***	55680	rs8045738	pros_gleason_prost	1.33 (1.08-1.62)	0.141	0.148	2.753	0.006	0.005
16q12.2	***LPCAT2***	0	rs10521319	pros_stage_m	1.11 (1.03-1.20)	0.000	0.057	2.619	0.009	0.009
		86077	rs9302667	pros_gleason_prost	1.35 (1.11-1.64)	0.156	0.170	2.994	0.003	0.003
		98065	rs893260	pros_gleason_prost	1.30 (1.07-1.58)	0.175	0.184	2.679	0.007	0.006
20p11.21	*GZF1*	52836	rs6076072	pros_stage	0.90 (0.84-0.97)	0.028	0.075	-2.914	0.004	0.003
		52836	rs6076072	pros_stage_t	2.17 (1.21-3.92)	0.068	0.035	2.589	0.010	0.009
20p13	*NSFL1C*	22006	rs6042568	pros_gleason	0.75 (0.62-0.90)	0.037	0.029	-3.007	0.003	0.004

The third paragraph of the section ‘Analysis of Human Prostate Cancer GWAS Data Reveals That QTL Candidate Gene SNPs Are Associated with Aggressive Prostate Cancer’ should read:

In the study, 1,372 SNPs mapped within a 100 kb radius of the 29 QTL candidate genes were tested in the CGEMS cohort. Analysis of aggressive vs. non-aggressive disease phenotypes were performed as per the comparisons described in Table 4. Correction for multiple testing was performed using permutation testing (n = 10,000 permutations). Fourteen of the 29 candidate genes exhibited evidence for association with clinical characteristics of aggressive prostate cancer (Table 5). Most notably, SNPs in three of the five genes associated with poor clinical outcomes in TCGA (Provisional) and GSE21032 prostate cancer gene expression datasets (*CXCL14*, *ITGAX*, and *LPCAT2*) were all associated with aggressive prostate cancer: For *CXCL14*, the following SNPs were associated with Gleason score at prostatectomy: rs2547 (permutation *P* = 0.003; OR = 0.61 [0.43–0.85]), rs2237061 (permutation *P* = 0.008; OR = 0.61 [0.43–0.88]), rs10515473 (permutation *P* = 0.002; OR = 0.72 [0.59–0.88]), and rs4463175 (permutation *P* = 0.003; OR = 1.42 [1.12–1.81]); and the following with metastasis to regional lymph nodes: rs801564 (permutation *P* = 0.010; OR = 1.05 [1.01–1.09]) and rs2067000 (permutation *P* = 0.004; OR = 1.05 [1.02–1.09]). For *ITGAX*, an association was apparent between rs8045738 and Gleason score at prostatectomy (permutation *P* = 0.005; OR = 1.33 [1.08–1.62]). Finally, for *LPCAT2*, rs10521319 was associated with distant metastasis (permutation *P* = 0.009; OR = 1.11 [1.03–1.20]), and rs9302667 (permutation *P* = 0.003; OR = 1.35 [1.11–1.64]) and rs893260 (permutation *P* = 0.006; OR = 1.30 [1.07–1.58]) with Gleason score at prostatectomy. Manhattan plots for all relevant genomic regions are shown in S5 Fig. Additionally, haplotypes in LD with these three QTL candidate genes were associated with clinical markers of prostate cancer aggressiveness (S15 Table).

## Supporting Information

S15 Table Statistically significant aggressive disease-associated haplotypes for QTL candidate genes in the CGEMS prostate cancer cohort. (XLSX)
