# Supplementary figures and images for: A Systems Genetics Approach Identifies CXCL14, ITGAX, and LPCAT2 as Novel Aggressive Prostate Cancer Susceptibility Genes
Source: PLoS Genet. 2014 Nov 20;10(11):e1004809. doi: 10.1371/journal.pgen.1004809 (PMC4238980; doi:10.1371/journal.pgen.1004809)

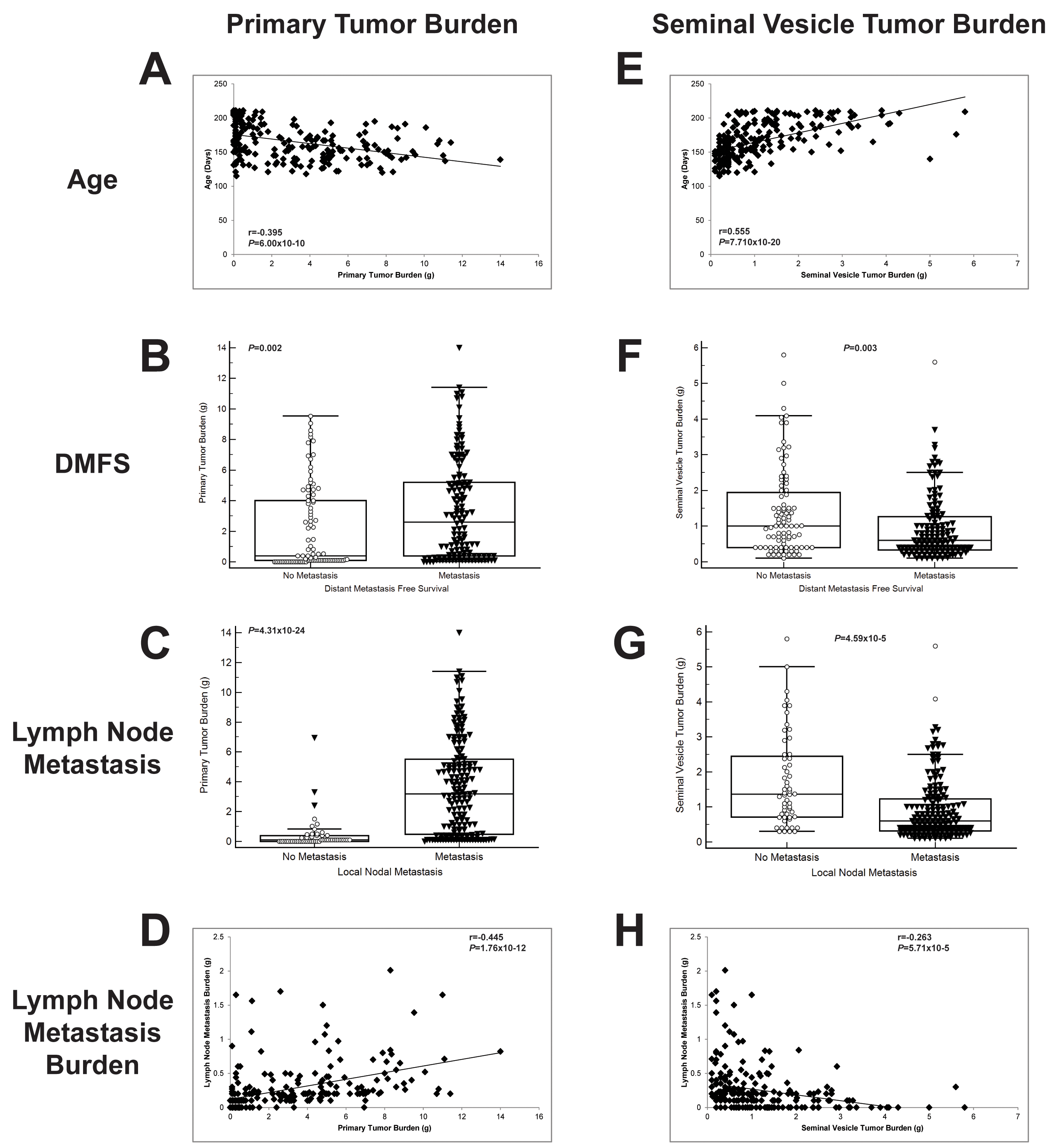

Supplement: Figure S1 — Correlations between tumor- and metastasis-related traits in (TRAMP × NOD/ShiLtJ) F2 mice. Primary prostate tumor burden exhibited a negative correlation with age of death (A) and positive correlations with DMFS (B), lymph node metastasis (C), and lymph node metastasis burden (D). Conversely, seminal vesicle tumor burden was positively correlated with age of death (E) and negatively correlated with DMFS (F), lymph node metastasis (G), and lymph node metastasis burden (H). (TIF) [file pgen.1004809.s001.tif]

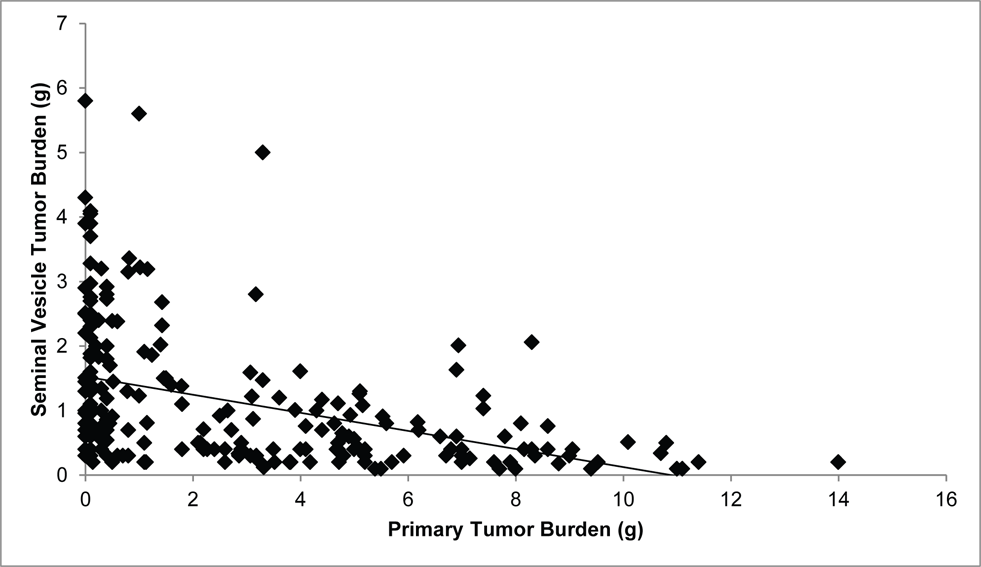

Supplement: Figure S2 — Correlation between seminal vesicle tumor burden and primary tumor burden in (TRAMP × NOD/ShiLtJ) F2 mice. (TIF) [file pgen.1004809.s002.tif]

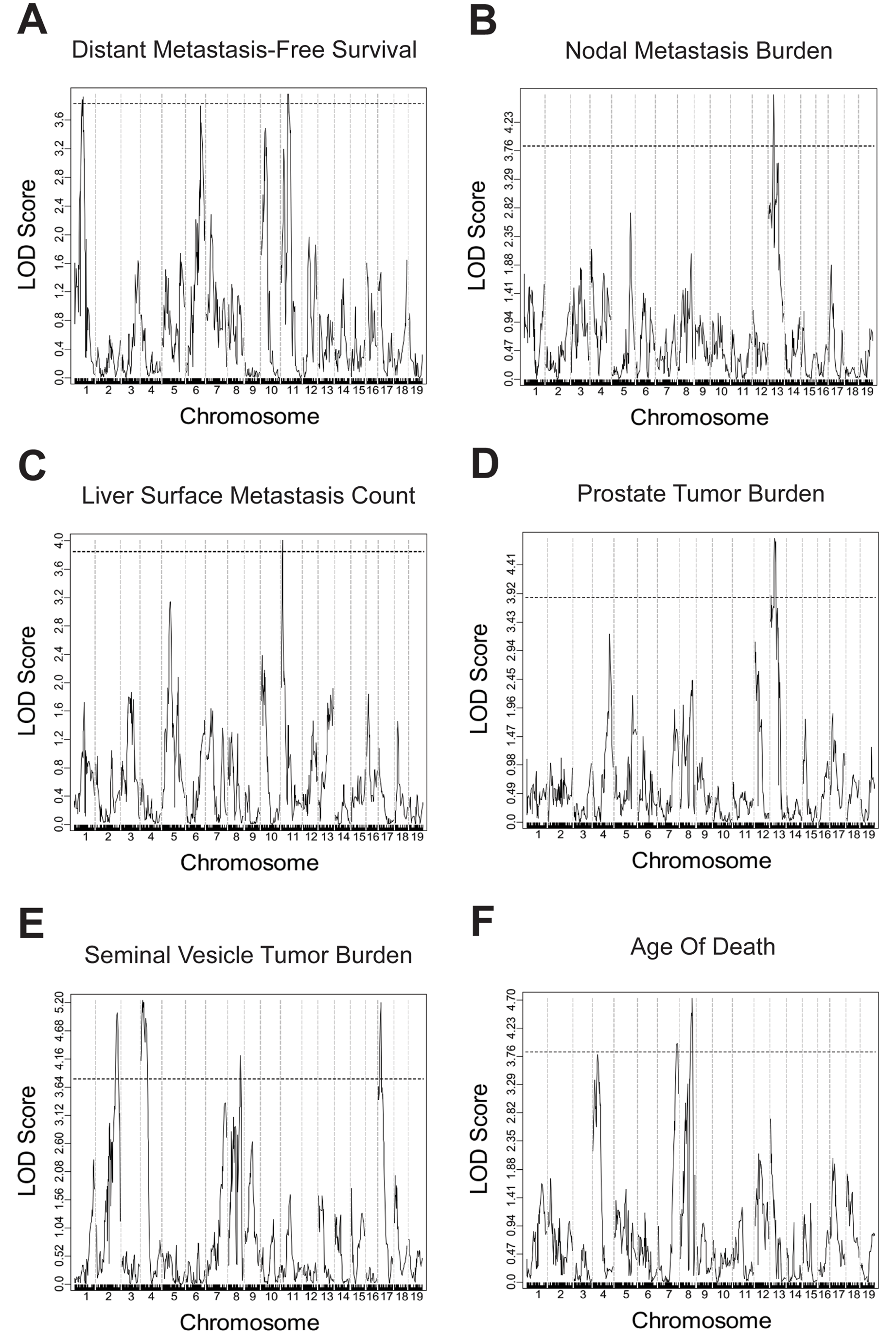

Supplement: Figure S3 — QTL plots for aggressive disease loci identified in (TRAMP × NOD/ShiLtJ) F2 mice. QTLs were observed for the following traits: (A) DMFS; (B) total nodal metastasis burden; (C) liver surface metastasis count; (D) prostate tumor burden; (E) seminal vesicle tumor burden; and (F) age of death. The horizontal dotted line represents a genome-wide level of statistical significance of α <0.05. (TIF) [file pgen.1004809.s003.tif]

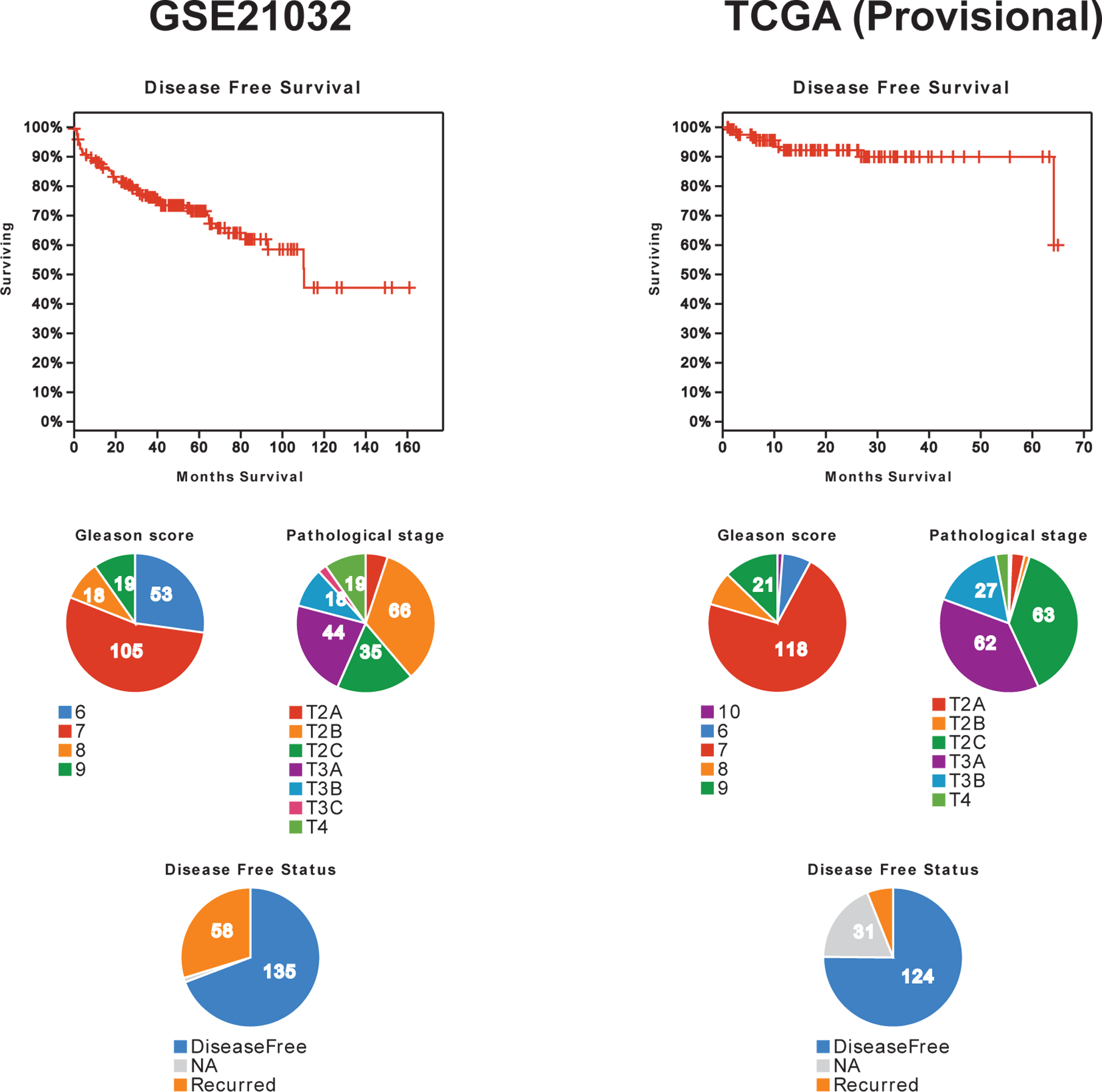

Supplement: Figure S4 — Clinical characteristics of patients represented in the GSE21032 and TCGA (Provisional) datasets. (TIF) [file pgen.1004809.s004.tif]

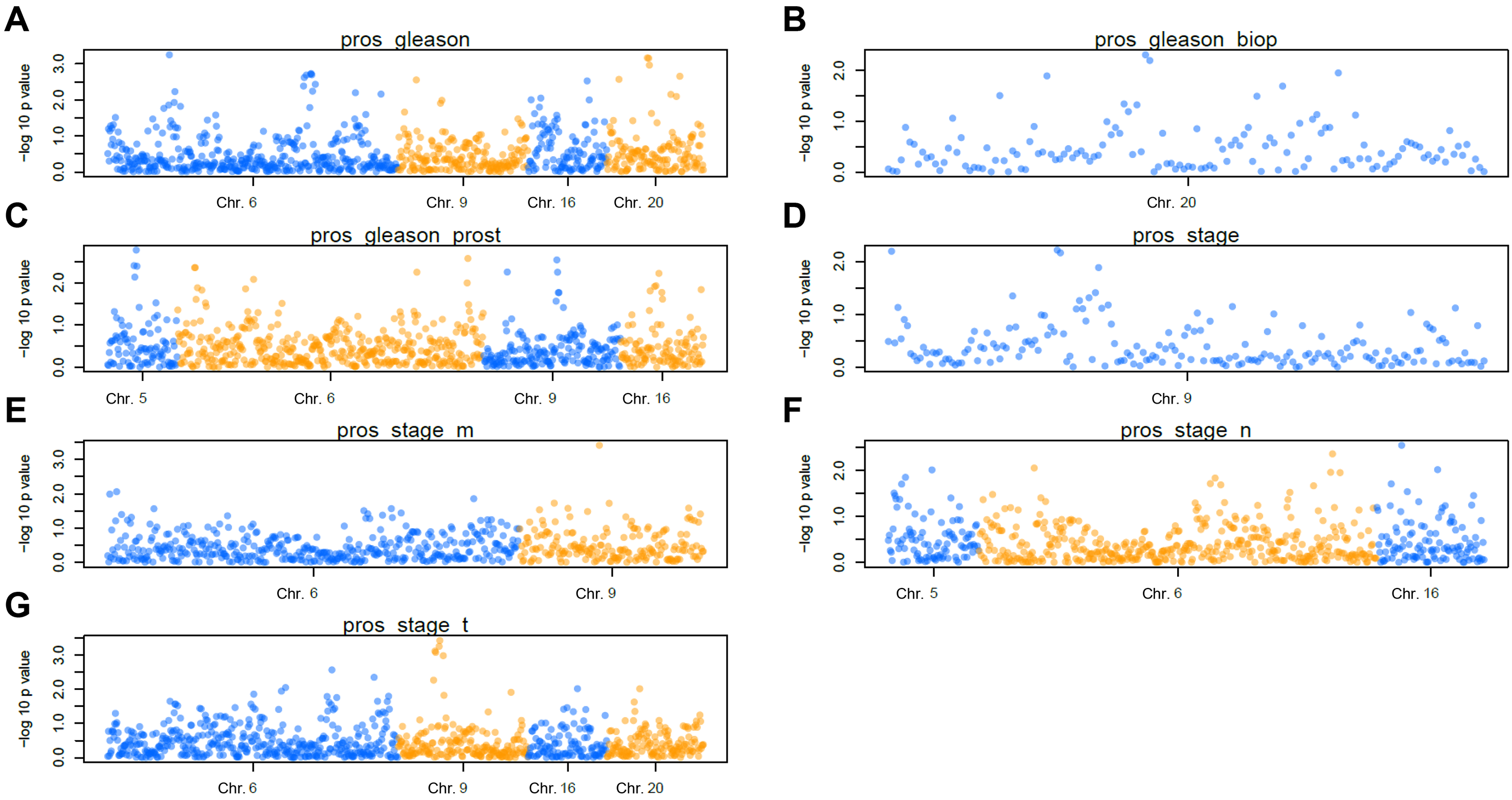

Supplement: Figure S5 — Manhattan plots for genomic regions of interest in CGEMS GWAS. Plots are only shown for regions where candidate gene SNPs were associated with the following phenotypes: (A) best Gleason score available; (B) biopsy Gleason score; (C) prostatectomy Gleason score; (D) prostate cancer stage; (E) distant metastasis; (F) nodal involvement; and (G) primary tumor stage. (TIF) [file pgen.1004809.s005.tif]
